# Simultaneous Determination of Phenolic Acids, Anthraquinones, Flavonoids, and Triterpenes of Cynomorii Herba in Different Harvest Times by LC-MS/MS

**DOI:** 10.1155/2020/8861765

**Published:** 2020-08-26

**Authors:** Hua Jin, Ge Tang, Jin Li, Lin Ma, Yuhong Li, Yan-xu Chang

**Affiliations:** ^1^College of Traditional Chinese Medicine, Tianjin University of Traditional Chinese Medicine, Tianjin 300193, China; ^2^Department of Nephrology, The First Teaching Hospital of Tianjin University of Traditional Chinese Medicine, Tianjin 300193, China; ^3^Tianjin State Key Laboratory of Modern Chinese Medicine, Tianjin University of Traditional Chinese Medicine, Tianjin 300193, China; ^4^College of Chinese Materia Medica, Tianjin University of Traditional Chinese Medicine, Tianjin 300193, China

## Abstract

A sensitive and accurate method was developed for the simultaneous determination of twelve components including phenolic acids (gallic acid, protocatechuic aldehyde, protocatechuic acid, and ferulic acid), flavonoids (catechin, epicatechin, rutin, luteolin, luteolin-7-glucoside, and epicatechin gallate), anthraquinones (emodin), and triterpenes (ursolic acid) in Cynomorii herba in different harvest times by liquid chromatography tandem mass spectrometry (LC-MS/MS). The chromatographic separation was achieved on an Eclipse plus C_18_ (3.0 mm × 50 mm, 1.8 *μ*m) column at 40°C. The mobile phase consisted of acetonitrile and 0.05% formic acid with a gradient elution at a flow rate of 0.4 mL·min ^−1^. Under the optimized conditions, there was good linear relation (*r* ≥ 0.9944) and satisfactory precision (RSD values less than 5.65%). The recoveries of the twelve components were in the range of 93.5–105%. Moreover, the limits of detection (LOD) ranged from 0.003 to 21 ng mL^−1^ for the twelve analytes. In conclusion, the validated method was successfully applied to analyze the change regularity of the twelve components of Cynomorii herba in different harvest times. It provides a theoretical basis for choosing the suitable harvesting time of Cynomorii herba.

## 1. Introduction

Cynomorii herba (Suoyang in Chinese, CH), one kind of traditional Chinese medicines, is the dried succulent stem of *Cynomorium songaricum* Rupr. It is an edible and medicinal plant that is widely distributed in the northwest desert of China, especially in Inner Mongolia, Ningxia, and Gansu province. It is usually used to make medicated wine and also used as food and fodder in the northwest region of China. CH has been widely used to improve sexual function, remedy intestinal ailments, postpone senility, nourish kidney, and treat impotence [1, 2]. Many pharmacological research studies revealed that CH could improve male fertility by enhancing spermatogenesis and glial-cell-derived neurotrophic factor (GDNF) expression. Moreover, the components of CH exhibited phytoestrogenic and phytoandrogenic activities, which were benefited for inhibition of oestrogen/androgen-induced BPH development [3–5]. Only one or several components were stipulated as a quality control marker of traditional Chinese medicine in Chinese Pharmacopoeia 2015. However, hundreds of components were existed in TCM, which may possess so many bioactivities. There was no components as a marker for CH in Chinese Pharmacopoeia 2015 [6]. However, the phytochemical investigation indicated that the main chemistry components of CH incorporate flavonoids, organic acids, polysaccharides, triterpenes, steroidal compounds, volatile oil, phenolic acids, steroids, alkaloids, and condensed tannins [7–9]. These components possessed many bioactivities including antitumor, antioxidant, antiaging, antibacterial activity, and the activities of inhibiting HCV protease and *α*-glucosidase [10–16]. Thus, it is necessary that multiple components were used for the quality control of CH.

A few analytical methods were established for analyzing chemical constituents of CH, such as thin-layer chromatography, capillary electrophoresis, high-performance liquid chromatography, and ultrahigh-performance liquid chromatography [17–20]. However, these methods suffered from low resolution, low sensitivity, or few analytes (less than five analytes). It was well known that the content of a single or a few compounds might not accurately reflect the quality of the complex herbal products [21]. Therefore, it was necessary to develop a simple and effective method to simultaneously determine multiple components for the quality control of CH.

Moreover, for herbal medicine, the contents of phytochemicals were usually influenced by many factors, such as producing area, harvest time, cultivation techniques, climatic conditions, drying method, and processing method. The constituents and contents of components changed with the harvest time [22, 23]. Therefore, choosing an appropriate harvesting time is the key to ensure the quality and efficiency of medicinal materials. In addition, the major source of CH is wild in traditional Chinese medicine. Due to the excessive collection and nonstandard collection methods, the wild resources of CH have decreased sharply in recent years. The artificial cultivation helped protecting the ecological environment and resource of CH. Thus, comparing the difference of components contents in wild and cultivated CH is necessary for ensuring the quality of medicinal materials.

**Figure 1:**
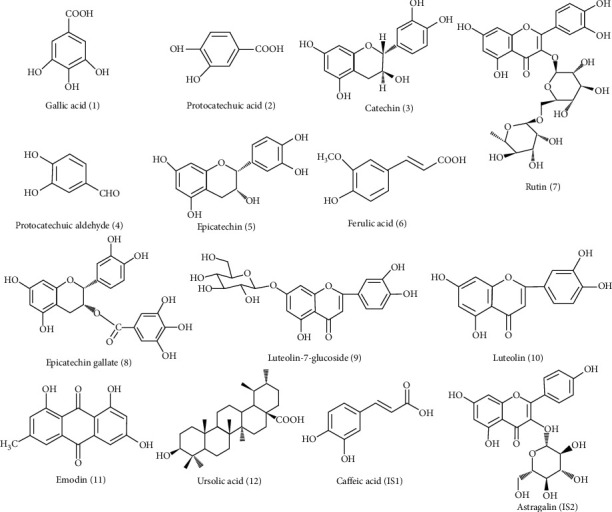
Chemical structures of twelve components and IS.

The aim of this study was to establish a rapid and sensitive LC-MS/MS method for simultaneous determination of phenolic acids (gallic acid, protocatechuic aldehyde, protocatechuic acid, and ferulic acid), flavonoids (catechin, epicatechin, rutin, luteolin, luteolin-7-glucoside, and epicatechin gallate), anthraquinones (emodin), and triterpenes (ursolic acid) in wild and cultivated CH samples in different harvesting seasons (Figure 1). A validated method can be used as a valid analytical method for intrinsic quality control of CH. Moreover, the quantitative analysis results can be helpful for choosing the best harvesting season of CH.

## 2. Materials and Methods

### 2.1. Materials and Reagents

Reference standards of gallic acid, caffeic acid (IS1), protocatechuic aldehyde, protocatechuic acid, ferulic acid, luteolin, luteolin-7-glucoside, catechin, epicatechin, emodin, and ursolic acid were purchased from the Chinese National Institute of Control of Pharmaceutical and Biological Products (Beijing, China). Epicatechin gallate, astragalin (IS2), and rutin were obtained from Chengdu Must Bio-Technology Co., Ltd. (Chengdu, China). The purity of all references is over 98.0%. Acetonitrile and formic acid of HPLC grade were achieved from Merck (Germany). The water with ultrapure grade was obtained from Milli-Q Water System (Millipore, USA). Other reagents were of analytical grade.

### 2.2. Herbal Plant

The Cynomorii herba, both wild and cultivated, were collected from Inner Mongolia province in China in different harvest times. The Cynomorii herba were identified by Professor Yan-xu Chang (Tianjin University of Traditional Chinese Medicine), and the voucher specimens were deposited at the Tianjin University of Traditional Chinese Medicine. First, the fresh samples were dried natural. Then, they were dried in oven at 105 °C until constant weight was obtained. Finally, these samples were smashed into powder using a pulverizer and passed through a 60-mesh sieve, which were prepared for the following experiment.

### 2.3. Chromatographic and Mass Spectrometric Conditions

The Agilent series 1200 HPLC system, which equipped with Agilent 6430 triple quadrupole mass spectrometer, was used for chromatographic analysis. All separations were carried out on an Agilent Eclipse plus C_18_ (3.0  mm × 50  mm i.d., 1.8 *μ*m) column. The mobile phases consisted of (*A*) aqueous formic acid (0.05%, v/v) and (B) acetonitrile. The gradient elution was as follows: 0–7 min, 10–25% B; 7–10 min, 25–90% B; and 10–15 min, 90–90% B at a flow rate of 0.4 mL·min ^−1^. The aliquot of 4 *μ*L was injected. In addition, the pressure of MS nebulizer was 35 psi. The flow rate and temperature of dried gas were 10 L min^−1^ and 325°C, respectively. The mass spectrometric parameters of all analytes were listed in Table 1.

### 2.4. Preparation of Internal Standard and Quality Control (QC) Samples

Caffeic acid and astragalin were dissolved with methanol to make a mixed internal standard (IS) solution at a concentration of 100 ng mL ^−1^. Each standard (10 mg) was dissolved by 10 mL methanol for preparing the twelve stock solutions. Quality control (QC) samples of the twelve components were prepared at medium concentration level by dissolving appropriate mixed standard solution in methanol, respectively. Then, a series of working standard solutions was achieved by mixing the twelve stock solution and diluted with methanol before use, including gallic acid, protocatechuic acid, catechin, protocatechuic aldehyde, epicatechin, ferulic acid, rutin, epicatechin gallate, luteolin-7-glucoside, luteolin, emodin, and ursolic acid in the range of 101–5050, 20.2–5050, 204–10200, 44.8–1120, 21.6–1080, 8.16–1020, 20.8–1040, 20.2–5050, 20.2–1010, 20.2–1010, 0.4–20, and 7.92–198 ng mL ^−1^, respectively. All solutions were stored at 4°C.

### 2.5. Sample Solution Preparation

The samples were powdered and passed through a 60-mesh sieve. The accurately weighed sample powder (0.100 g) was ultrasonic extracted with 10 mL of 70% (v/v) methanol for 20 min, cooled at room temperature, and subsequently centrifuged for 10 min at 14000 rpm. Then, IS (10 *μ*L) was added to the supernatant (990 *μ*L). The final solutions were vortexed for 30 seconds and centrifuged for 10 min. 4 *μ*L of the supernatant was injected into the HPLC.

### 2.6. Method Validation

These analytes of standard stock solutions were diluted to a series of appropriate concentrations for plotting the calibration curves. Then, the curves were constructed by plotting the peak areas versus the concentration of each analyte.

The QC samples at three levels of different concentration were applied to evaluate the accuracy and precision in same day (intraday) and between three different days (interday). The assessed index was RSD and the percent ratios of the calculated concentration to nominal concentration. The range of accuracy should be 95.0 to 105%.

Stability study was performed with sample solution in 24 h (the time-point is 0, 2, 4, 6, 8, 12, and 24 h, respectively) at the condition of temperature. The repeatability was investigated by using the developed method parallelly six times.

The recovery was tested by adding mixed reference standard solution to the untreated sample to yield final concentration. The sample was processed by the sample preparation procedure (*n* = 6).

## 3. Results

### 3.1. Internal Standard (IS) Selection

In the study, the four kinds of compounds were simultaneously determined. Caffeic acid and astragalin were chosen as internal standard for phenolic acid, flavonoid, anthraquinone, and triterpene and no interference from endogenous substances.

### 3.2. Optimization of Extraction Conditions

In order to achieve an optimum extraction of target components in CH, the key factors such as methanol concentration (50, 70 and 90%, v/v) and ultrasonic time (20, 30 and 40 min) were investigated by using combination experiment, while sample-solvent ratio (1 : 100, w/v) was kept constant. Total concentration of the twelve target components in the extract solution was selected as the evaluating indicator (Table 2). It was found that total concentration of the twelve target components in the extract solution was 6323.8 ng/mL when 70% (v/v) methanol was selected to extract the target components for 20 min in ultrasonic extraction process. Finally, the optimum condition was obtained when the sample was extracted by 70% (v/v) methanol for 20 min.

### 3.3. Optimization of HPLC-MS/MS Conditions

To obtain good chromatographic separation, various HPLC parameters including mobile phase modifier (0, 0.05, 0.1, and 0.2% formic acid), column temperature (35, 40, and 45°C), and flow rate (0.3, 0.4 and 0.5 mL·min ^−1^) were also investigated in detail for analyzing the target compounds. Take into consideration the peak response and separation, the optimum HPLC conditions were obtained when mobile phase modifier was 0.05% formic acid, column temperature was 40 °C, and flow rate was 0.4 mL·min ^−1^.

Twelve components were identified by comparing their retention time and the MS information with those of reference standards, respectively. The results showed that the highest sensitivity was obtained at a certain value of collision energy (CE).

### 3.4. Method Validation

#### 3.4.1. Specificity

**Figure 2:**
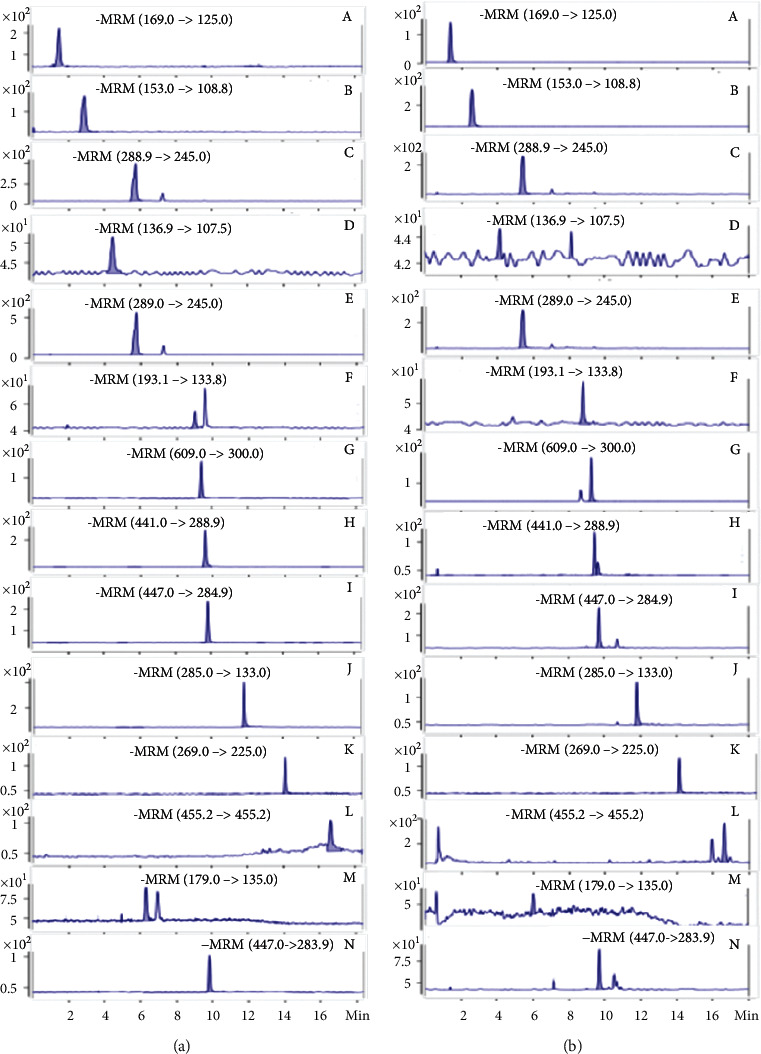
Representative chromatograms of (a) mixed standard solution and (b) the real sample solution of gallic acid (A), protocatechuic acid (B), catechin (C), protocatechuic aldehyde (D), epicatechin (E), ferulic acid (F), rutin (G), epicatechin gallate (H), luteolin-7-glucoside (I), luteolin (J), emodin (K), ursolic acid (L), caffeic acid (M), and astragalin (N).

Figure 2 showed the chromatograms of twelve compounds of CH in the MRM mode. The results demonstrated that there was good separation and no interference from endogenous components for all analytes.

#### 3.4.2. Precision and Stability

The accuracy was achieved by evaluating QC samples of twelve components in one day (*n* = 6). The accuracies of twelve components for intraday and interday within range of 87.2–107.3%. The RSD value was below 5.65% (Table 3). These results demonstrated that the developed method was accurate and reproducible.

The stabilities of the twelve components were determined at 0 h, 2 h, 4 h, 6 h, 8 h, 12 h, and 24 h, respectively. As present in Table 3, the RSD values were below 8.98%. The results indicated that there was good stability within 24 h for all analytes.

#### 3.4.3. Calibration Curves, Limit of Detection (LOD), Limit of Quantification (LOQ), Repeatability, and Recovery

Under the optimum chromatographic conditions, all calibration curves of the twelve components were achieved with wide concentration ranges and the correlation coefficients of them were greater than 0.9944. For the twelve components, the LOD ranged from 0.003 to 21 ng mL^−1^, and the limit of LOQ ranged from 0.01 to 18 ng mL^−1^. RSD values of the repeatability were less than 9.10%. These results were shown in Table 4.

The recovery was performed for evaluating the precision and accuracy of the established method. The recoveries of twelve compounds were in range of 93.5–105.0%, and their RSD values were less than 6.63% (Table 4). The above results indicated that the developed method possessed good accuracy and precision.

### 3.5. Analysis of Four Kinds of Components (Phenolic Acid, Flavonoid, Anthraquinone, and Triterpene) at Different Harvest Times and from Different Sources

The established method was applied to simultaneously determine the twelve components of CH at four different harvesting times and from two different sources (wild and cultivated). The variation of contents of twelve components in different harvest times and sources were shown in Table 5.

For different sources, the results revealed that the total contents of the four kinds of components including phenolic acids, flavonoids, anthraquinones, and triterpenes in the cultivated sample were higher than those of the four kinds of components in the wild sample in four harvesting times. Therefore, cultivated CH can be used instead of wild CH. Artificial cultivation has solved the problems such as resource scarcity and destruction of ecological environment for CH.

For different harvest times, the contents of phenolic acids (protocatechuic acid, catechin, and ferulic acid) and the total phenolic acid were the highest in fructicative period. Compared with the other periods, the content of gallic acid was higher in pre-unearth period. Emodin (anthraquinones) was detected only in the sample at fructicative period. Moreover, the content of ursolic acid (triterpenes) was highest during fructicative period. These results indicated that the fructicative period could be the optimum harvest time for anthraquinones and triterpenes. In addition, the contents of flavonoids (epicatechin gallate, luteolin-7-glucoside, luteolin) were highest in pre-unearth period. The contents of protocatechuic aldehyde, epicatechin, and total flavonoids were higher in unearth period.

To sum up, for the three kinds of components (phenolic acid, anthraquinones, and triterpenes), the productive period was the optimum harvest time. However, the unearth period was the appropriate harvest time for flavonoids. Because *Cynomorium songaricum* is a parasitic plant, its standardized cultivation is difficult. At present, *Cynomorium songaricum* was only cultivated in one area in Inner Mongolia of China. In the future, more cultivated and wild samples with different harvesting times should be analyzed to support the results and conclusion by the established LC-MS/MS method.

## 4. Conclusions

A simple and effective method was developed for the simultaneous determination of the twelve components in wild and cultivated Cynomorii herba in different harvest times. The results indicated that the established method possessed excellent sensitivity and selectivity. Moreover, the application of this method could successfully evaluate the quality of wild and cultivated CH. It is crucial for solving the problem of resource scarcity.

In addition, the contents of the four kinds of components varied with the different harvest period. We can choose the optimum harvest time according to the requirements of different types of chemical ingredients. Consequently, the developed HPLC-MS/MS method provided the theoretic basis for quality control and resource protection of CH.

## Figures and Tables

**Table 1:** Mass spectrometric parameters for all analytes.

Compounds	Precursor ion	Product ion	Fragmentor	CE
Gallic acid	169.0	125.0	90	8
Protocatechuic acid	153.0	108.8	75	9
Catechin	288.9	245.0	140	10
Protocatechuic aldehyde	136.9	107.5	100	19
Epicatechin	289.0	245.0	145	10
Ferulic acid	193.1	133.8	75	10
Rutin	609.0	300.0	235	39
Epicatechin gallate	441.0	288.9	150	10
Luteolin-7-glucoside	447.0	284.9	210	25
Luteolin	285.0	133.0	160	33
Emodin	269.0	225.0	140	21
Ursolic acid	455.2	455.2	115	0
Caffeic acid (IS1)	179.0	135.0	90	10
Astragalin (IS2)	447.0	283.9	180	25

**Table 2:** The effect of the different extract conditions on the target components (ng/mL).

Extract condition	Gallic acid	Protocatechuic acid	Catechin	Protocatechuic aldehyde	Epicatechin	Ferulic acid	Rutin	Epicatechin gallate	Luteolin-7-glucoside	Luteolin	Emodin	Ursolic acid	Total concentrations
50%–20 min	1346	773	490	51	431	18	121	1052	477	68	5.7	27	4859.7
50%–30 min	1435	852	508	96	446	21	152	906	509	52	1.8	26	5004.8
50%–40 min	1803	949	557	91	487	19	141	1282	499	50	1.0	37	5916.0
70%–20 min	1899	942	641	98	559	18	170	1347	538	51	0.8	60	6323.8
70%–30 min	1683	914	591	122	517	18	159	1086	505	42	0.7	56	5693.7
70%–40 min	1581	938	589	75	515	17	144	1083	504	46	0.5	59	5551.5
90%–20 min	1224	843	533	118	467	15	106	569	355	35	0.5	61	4326.5
90%–30 min	1381	960	595	113	520	20	144	748	452	42	0.5	68	5043.5
90%–40 min	1541	936	603	124	527	21	126	966	444	44	0.4	67	5399.4

*Note.* The sample powder (0.100 g) was ultrasonic extracted with 10 mL methanol solution at different concentrations.

**Table 3:** Precision and stability of twelve components (*n* = 6).

Components	Concentration (ng·mL^−1^)	Intraday		Interday		Stability (*n* = 6)	
		RSD (%)	Accuracy (%)	RSD (%)	Accuracy (%)	RSD (%)	Accuracy (%)
Gallic acid	505	3.1	103.7	0.1	103.7	3.08	96.6
Protocatechuic acid	505	3.5	103.9	0.6	104	2.38	101.5
Catechin	5100	3.1	103.2	1.8	101.1	3.70	94.6
Protocatechuic aldehyde	560	2.4	103.3	1.7	104.2	5.34	102.8
Epicatechin	540	2.4	104.8	1.6	104.2	3.54	95.3
Ferulic acid	102	2.6	100.5	3.2	98.7	3.43	103.0
Rutin	104	4.2	103.4	2.2	102.6	5.83	96.1
Epicatechin gallate	505	2.6	104.5	2.0	102.9	8.32	82.6
Luteolin-7-glucoside	101	3.2	103.7	1.2	102.9	4.70	104.9
Luteolin	100	3.0	102.7	2.9	101.3	8.98	93.6
Emodin	2	4.4	101.1	1.2	102.0	4.53	110.8
Ursolic acid	99	3.1	103.3	0.5	103.3	5.58	105.9

**Table 4:** Calibration curves, LOD, LOQ, Repeatability, and Recovery of all analytes (*n* = 6).

Components	Linear regression	Linear range (ng·mL^−1^)	*r * ^2^	LOD (ng·mL^−1^)	LOQ (ng·mL^−1^)	Repeatability RSD (%)	Recovery	
							Mean (%)	RSD (%)
Gallic acid	*Y* = 0.2895*X* + 0.0354	101–5050	0.9994	5	15	5.75	94.0	3.08
Protocatechuic acid	*Y* = 0.1929X − 0.0040	20.2–5050	0.9994	6	18	4.32	95.5	4.00
Catechin	*Y* = 0.1018*X* + 0.0280	204–10200	0.9998	6	18	5.48	101.6	6.43
Protocatechuic aldehyde	*Y* = 0.0122*X* + 0.0034	44.8–1120	0.9998	1.5	4	3.71	100.5	5.04
Epicatechin	*Y* = 0.9618*X* + 0.0280	21.6–1080	0.9998	7	21	3.11	97.7	5.12
Ferulic acid	*Y* = 0.1414X − 2.2003e-004	20.2–1020	0.9995	3	10	7.36	99.3	5.53
Rutin	*Y* = 0.9379X − 0.0038	20.8–1040	0.9996	0.5	1.5	7.86	98.1	5.83
Epicatechin gallate	*Y* = 0.3410X − 0.0124	20.2–5050	0.9994	1	3	6.93	97.7	6.63
Luteolin-7-glucoside	*Y* = 1.6412*X* + 0.0779	20.2–1010	0.9998	0.1	0.3	2.64	105.0	4.28
Luteolin	*Y* = 21.2695*X* + 17.0019	20.2–1010	0.9990	0.1	0.3	2.35	102.5	3.71
Emodin	*Y* = 5.3529*X* + 0.0097	0.4–20	0.9997	0.003	0.01	0.00	104.3	2.24
Ursolic acid	*Y* = 0.1071*X* + 1.1690	7.92–198	0.9944	0.3	1.0	9.10	93.5	1.43

**Table 5:** Quantitative analytical results (*n* = 3).

Source	Harvesting times	Mean contents of compounds 1–12 (*μ*g g^−1^)															
		Phenolic acids					Flavonoids							Anthraquinones		Triterpenes	
		1	2	3	6	Total	4	5	7	8	9	10	Total	11	Total	12	Total
Cultivated	Pre-unearth period	25.287	17.815	4.575	0.645	48.322	100.364	10.626	0.480	8.574	6.725	0.473	127.774	0	0	1.873	1.873
	Unearth period	12.305	22.674	5.231	0.966	41.176	128.430	13.598	0.495	5.003	1.707	0.099	149.894	0	0	1.890	1.890
	Flowering period	12.134	22.455	10.233	0.678	45.500	58.809	6.227	0.585	2.829	0.842	0.090	70.442	0	0	2.031	2.031
	Fructicative period	21.095	29.393	10.725	2.608	63.821	48.740	5.161	1.087	3.525	0.886	0.139	59.867	0.0133	0.0133	2.409	2.409

Wild	Pre-unearth period	17.7035	12.7555	6.0535	0.7875	37.3	83.425	8.834	0.322	1.2105	1.572	0.0955	95.966	0	0	1.55	1.55
	Unearth period	14.103	23.22	4.9295	0.361	42.6135	81.847	8.666	0.457	1.6785	0.3025	0.0535	93.533	0	0	1.835	1.835
	Flowering period	14.462	22.908	3.546	0.229	41.145	7.833	0.829	0.365	0.728	0.266	0.056	10.384	0	0	1.551	1.551
	Fructicative period	14.714	14.043	1.897	4.534	35.188	53.972	5.715	0.721	2.745	0.531	0.116	63.979	0.0003	0.0003	1.982	1.982

Note: gallic acid (1), protocatechuic acid (2), catechin (3), protocatechuic aldehyde (4), epicatechin (5), ferulic acid (6), rutin (7), epicatechin gallate (8), luteolin-7-glucoside (9), luteolin (10), emodin (11), and ursolic acid (12).

## Data Availability

The data used to support the ﬁndings of this study are available from the corresponding author upon request.
